# Excess Deaths Associated with Rheumatic Heart Disease, Australia, 2013–2017

**DOI:** 10.3201/eid3001.230905

**Published:** 2024-01

**Authors:** Ingrid Stacey, Rebecca Seth, Lee Nedkoff, Vicki Wade, Emma Haynes, Jonathan Carapetis, Joseph Hung, Kevin Murray, Dawn Bessarab, Judith Katzenellenbogen

**Affiliations:** The University of Western Australia, Perth, Western Australia, Australia (I. Stacey, L. Nedkoff, E. Haynes, J. Carapetis, J. Hung, K. Murray, D. Bessarab, J. Katzenellenbogen);; Curtin University, Perth, Western Australia (R. Seth);; Victor Chang Cardiac Research Institute, Darlinghurst, New South Wales, Australia (L. Nedkoff);; National Heart Foundation of Australia, East Sydney, New South Wales, Australia (V. Wade);; Telethon Kids Institute, Nedlands, Western Australia, Australia (J. Carapetis, J. Katzenellenbogen)

**Keywords:** Rheumatic heart disease, *Streptococcus pyogenes*, epidemiology, cardiovascular diseases, mortality, excess deaths, bacteria, Australia

## Abstract

During 2013–2017, the mortality rate ratio for rheumatic heart disease among Indigenous versus non-Indigenous persons in Australia was 15.9, reflecting health inequity. Using excess mortality methods, we found that deaths associated with rheumatic heart disease among Indigenous Australians were probably substantially undercounted, affecting accuracy of calculations based solely on Australian Bureau of Statistics data.

Rheumatic heart disease (RHD), caused by *Streptococcus pyogenes* infections, is driven by social determinants of health and disproportionately affects Aboriginal and Torres Strait Islanders in Australia (hereafter Indigenous Australians), causing premature illness and death (*1*–*3*). Deaths associated with RHD can be prevented by addressing poor living conditions, treatment delays, racism, and healthcare inaccessibility (*2*,*4*–*6*). Approximately 663 deaths associated with RHD among Indigenous Australians are predicted for 2016–2031 (*7*). Our previous analysis of persons from 5 jurisdictions in Australia who had RHD, were <65 years of age, and died during 2013–2017 (covering 86% of the Indigenous population) revealed that RHD was the underlying cause of death for only 15.0%; cause of death was recorded as underlying noncardiovascular for 42.7%, and cause of death among Indigenous Australians was missing for 13.7% (*2*). Thus, the burden of death associated with RHD is potentially underestimated when measured by using RHD-coded death records from the Australian Bureau of Statistics (ABS). Concerns regarding inaccurate or missing cause-of-death data can be reduced by using excess mortality methods, which measure deaths directly and indirectly attributable to RHD (*8*). Consequently, we used excess mortality methods, independent of ABS RHD-coded records, to estimate RHD-associated deaths for 2013–2017 in Australia.

## The Study

**Figure 1:**
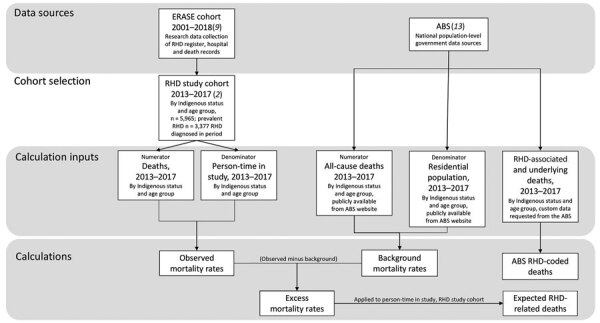
Data sources, cohort selection, and calculations generated in study of excess deaths associated with rheumatic heart disease, Australia, 2013–2017 (*2*,*9*,*10*). The main study outputs are observed mortality rates, excess mortality rates, and expected RHD-associated deaths (bottom row). ABS, Australian Bureau of Statistics; RHD, rheumatic heart disease.

In a cross-sectional study, we used linked administrative health and ABS data to estimate RHD-related deaths (Figure 1). We estimated observed mortality rates by age at death and Indigenous status by using data from End RHD in Australia: Study of Epidemiology (ERASE) (*9*). We used the generated excess deaths rates to calculate expected RHD-associated deaths and compared them with ABS RHD-coded death counts.

The ERASE cohort has been described (*2*). In brief, prevalent and new RHD cases were identified from the RHD register, surgical registry, and hospitalization records (*1*,*9*,*11*,*12*). ERASE included 5 jurisdictions in Australia: Northern Territory, Queensland, South Australia, Western Australia, and New South Wales (Appendix Figure 1). We obtained probabilistically linked data from jurisdiction-specific linkage units; ERASE investigators harmonized variables between jurisdictions and data sources and determined vital status.

To create the RHD study cohort, we selected ERASE cohort members who had RHD, were <65 years of age, and were alive on January 1, 2013 (Figure 1). We used broad age groups (0–24, 25–44, and 45–64 years), which corresponded to those used in previous RHD mortality studies (*2*,*13*). We used multiple ERASE data sources to assign Indigenous status, minimizing known underidentification (*9*). We searched all hospitalization record diagnosis fields for comorbidities (Appendix Table 1).

We calculated observed and background mortality rates (both crude and age-specific per 100,000 person-years). We calculated age-standardized mortality rates by using the direct method, standardized to World Health Organization World Standard Population 5-year age groupings for 2000–2025. For observed mortality rates (Figure 1), RHD diagnoses from January 1, 2013, through December 31, 2017, contributed person-time from whichever time was latest (denominators): first diagnosis date or January 1, 2013. Deaths during 2013–2017 contributed to observed mortality rate numerators. For background mortality rates (Figure 1), we used age group–specific deaths of Indigenous and non-Indigenous Australians (numerators) and residential population denominators from the ABS (*13*).

We calculated excess mortality rates as the difference between the observed and background mortality rates (within matched age/population stratum; Figure 1). We derived 95% CIs by using nonparametric bootstrap methods, assuming a Poisson distribution (Appendix). We calculated expected RHD-related deaths by applying excess mortality rates to person-years within the RHD study cohort age/population stratum (Appendix Table 2). We calculated observed and excess mortality rate ratios (MRRs) with 95% CIs by comparing Indigenous with non-Indigenous populations with RHD.

Epidemiologic, demographic, and clinical characteristics of this cohort are described (Appendix Table 3). Among the 9,342 persons in the RHD study cohort (65.6% female, 24.6% <25 years of age, 55.6% Indigenous), comorbidities included atrial fibrillation (30.5%), heart failure (26.0%), hypertension (23.7%), diabetes (19.4%), chronic kidney disease (17.4%), and chronic obstructive pulmonary disease (10.6%) (Appendix Table 3). The 726 observed cohort deaths occurred most frequently among persons 45–64 years of age (72.3%) and among those who were female (58.7%) (Appendix Table 3). Among the 325 non-Indigenous persons who died, 36.0% were immigrants from low/middle income countries. Metropolitan residents accounted for 14.0% (n = 56) of deaths among Indigenous and 71.4% (n = 232) among non-Indigenous persons. Detailed causes of death within the study cohort were attributed to mostly noncardiovascular causes; most frequent were cancer, diabetes mellitus, and respiratory diseases (*2*).

**Figure 2:**
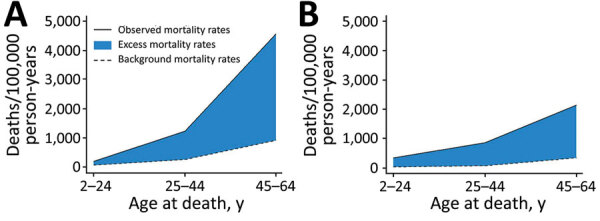
Excess RHD-associated mortality by Indigenous status and age at death, Australia, 2013–2017. A) Indigenous; B) non-Indigenous. Background mortality rates (from the Australian Bureau of Statistics) were subtracted from the observed mortality rates (in the RHD study cohort), generating excess mortality rates (the direct and indirect RHD-associated mortality rate). RHD, rheumatic heart disease.

In 2013–2017 in Australia, the background mortality rate was 193.6 deaths/100,000 Indigenous person-years and 72.3 deaths/100,000 non-Indigenous person-years (Table). Background age-specific mortality rates increased with advancing age in both populations but were always 2- to 3-fold higher for the Indigenous than non-Indigenous population (Table, Figure 2)

**Table:** Mortality rates associated with RHD among persons <65 years of age, Australia, 2103–2017*

Age group, y	Indigenous			Non-Indigenous			Rate ratio (95% CI)
	No.	Rate (95% CI)		No.	Rate (95% CI)		
Background mortality rates†							
0–24	1,319	70.4 (66.7–74.4)		8282	32.6 (32.0 –33.4)		2.16 (2.03–2.29)
25–44	2,302	262.87 (252.2–273.8)		17,004	74.20 (73.1– 75.3)		3.54 (3.29–3.60)
45–64	5,221	921.66 (896.8 –947.0)		69,752	344.26 (341.7–346.8)		2.68 (2.60–2.75)
Crude, 0–64	8,842	266.83 (261.3– 272.4)		95,038	138.64 (137.8–139.5)		1.92 (1.88–1.97)
ASMR, 0–64	8,842	193.55 (189.5– 197.6)		95,038	72.29 (71.8– 72.7)		2.68 (2.66–2.70)
Observed mortality rates‡							
0–24	13	204.1 (93.2–315.1)		<5	339.4 (0–723.4)		NC
25–44	112	1,238.3 (1,009.0–1,467.7)		34	858.4 (569.8–1,146.9)		1.44 (0.98–2.12)
45–64	276	4,568.6 (4,029.6–5,107.6)		288	2,140.1 (1,892.9–2,387.3)		2.13 (1.81–2.52)
Crude, 0–64	401	1,869.1 (1,687.9–2,050.4)		325	1,775.7 (1,584.4–1,967.1)		1.05 (0.91–1.22)
ASMR, 0–64	401	1,451.6 (1,307.0–1,596.2)		325	883.6 (674.3–1,092.9)		1.64 (1.42–1.9)
Excess mortality rates§							
0–24	9¶	136.7 (39.3–249.0)		<5¶	308.40 (0–751.3)		NC
25–44	88¶	1,000.2 (786.4–1230.9)		29¶	760.7 (487.8–1,047.3)		1.31 (1.02–2.31)
45–64	222¶	3,720.4 (3,184.8–4305.8)		240¶	1,817.8 (1,566.9–2,066.8)		2.05 (1.81–2.5)
Crude, 0–64	319¶	1,636.7 (1,459.8–1822.1)		272¶	1,646.7 (1,454.5–1,847.4)		0.99 (0.85–1.16)
ASMR, 0–64	319¶	1,166.0 (1,028.8–1317.6)		272¶	770.8 (584.3–989.0)		1.51 (1.14–2.02)
ABS RHD-coded mortality rates#							
0–24	8	0.43 (0.13–0.72)		7	0.03 (0.01–0.05)		15.49 (5.62–42.71)
25–44	48	5.48 (3.93–7.03)		41	0.18 (0.12–0.23)		30.64 (20.19–46.48)
45–64	89	15.71 (12.45–18.98)		252	1.24 (1.09–1.40)		12.63 (9.92–16.09)
Crude, 0–64	145	4.38 (3.66–5.09)		300	0.44 (0.39–0.49)		10.00 (8.20–12.19)
ASMR, 0–64	145	5.25 (4.40–6.11)		300	0.33 (0.29–0.37)		15.85 (13.00–19.33)

*Rates are deaths/100,000 person-years; intervals for excess mortality rates were obtained from bootstrapping of estimates (interpreted as 95% CIs). ABS, Australian Bureau of Statistics; ASMR, age-standardized mortality rate; NC, not calculated (numbers too low for reliable estimate); RHD, rheumatic heart disease. †Population level, n = 14,372,851. ‡Deaths from all causes within the RHD study cohort (n = 9,342). §Observed mortality rates minus background mortality rates. ¶Expected number of deaths associated with RHD were calculated on the basis of excess mortality rate applied to person-years within appropriate age/population strata. #Whole population. Previously published data, reproduced with permission (*2*). Population-level mortality rates based on ABS RHD-coded data (RHD as an underlying or associated cause of death).

In the RHD study cohort, 401 Indigenous and 325 non-Indigenous persons died, corresponding to observed mortality rates of 1,451 deaths/100,000 Indigenous person-years and 883 deaths/100,000 non-Indigenous person-years (Table). Age-specific mortality rates among Indigenous persons were highest among those 45–64 years of age (4,568 deaths/100,000 person-years; Figure 2); corresponding MRR was 2.13 (95% CI, 1.81–2.52) for Indigenous versus non-Indigenous persons (Table).

For the RHD study cohort, we estimated excess mortality rates of 1,166 deaths/100,000 Indigenous person-years and 771 deaths/100,000 non-Indigenous person-years, generating an MRR of 1.5 (Table). Excess mortality rates were highest among Indigenous persons 45–64 years of age for whom the peak excess MRR of 2.1 was observed (Table; Figure 2). Excess mortality rates applied to RHD study cohort strata estimated that 319 Indigenous and 272 non-Indigenous deaths were directly or indirectly associated with RHD (Table; Appendix Table 4). By comparison, ABS RHD-coded deaths captured 145 Indigenous deaths, less than half the expected cases (174 fewer than expected), but 300 non-Indigenous deaths, approximately the same as expected (28 more).

Accuracy of our estimates is limited by the quality of the coded information within source datasets and constrained by available data, including nonavailability of migrant population denominator information for rate calculations. The RHD mortality rates that we report also do not capture the profound effects that those deaths had on families, communities, and cultures.

## Conclusions

After adjusting for background mortality in Indigenous and non-Indigenous populations, we found that excess deaths were higher among persons with RHD. The excess mortality method applied to the RHD study cohort estimates both direct and indirect RHD-associated deaths and reduces concerns regarding misclassified and missing cause of death arising from use of ABS RHD-coded data only. Our method is particularly useful with the Indigenous population, for whom missing ABS RHD-coded data are an issue. RHD is probably not the only underlying driver of observed excess premature deaths; rather, RHD is a potent marker of the inequities experienced by Indigenous Australians and drives excess deaths indirectly in synergy with other chronic health conditions associated with social determinants. Expected deaths among non-Indigenous persons corresponded closely to ABS RHD-coded records; however, among the Indigenous population, excess deaths were more than twice those recorded in ABS (*2*). Similar to other chronic illnesses (diabetes and dementia [*10**,**14*]), the burden of RHD-associated deaths in Australia is potentially underascertained when based exclusively on ABS RHD-coded records, especially among Indigenous persons, for whom cause-of-death data are missing for >10% and multiple comorbidities, along with underlying RHD, contribute to death (*2*). Before Australia can achieve its RHD elimination goals, improved quality of Indigenous cause-of-death data and identification of synergistic factors contributing to excess RHD-associated deaths are needed (*7*).

Appendix Additional information for study of excess deaths associated with rheumatic heart disease, Australia, 2013–2017.
