# Supplementary material for: Excess Deaths Associated with Rheumatic Heart Disease, Australia, 2013–2017
Source: Emerg Infect Dis. 2024 Jan;30(1):146–50. doi: 10.3201/eid3001.230905 (PMC10756360; doi:10.3201/eid3001.230905)
Supplement: Appendix — Additional information for study of excess deaths associated with rheumatic heart disease, Australia, 2013–2017. [file 23-0905-Techapp-s1.pdf]

Article DOI: https://doi.org/10.3201/eid3001.230905

EID cannot ensure accessibility for supplementary materials supplied by authors. Readers who have difficulty accessing supplementary content should contact the authors for assistance.

Excess Deaths Associated with Rheumatic Heart Disease, Australia, 2013–2017

Appendix

Overview of bootstrap method for generation of confidence intervals (implemented in R) (*1*)

The RHD study cohort,

$X = \{x_{S1}, x_{S2}, \ldots, x_{SN_s}\}$,

where S was the stratum defined by age group and Indigenous/non-Indigenous combinations, and N_S was the number of observations within stratum S was randomly sampled with replacement within each of the six strata.

Specifically:

1. Strata-specific bootstrap samples were generated of size N_S
2. For each bootstrapped sample, strata-specific estimates of the following were generated:

For the age and population strata:

$$Excess\ mortality\ rate = \left(\frac{\sum_{n=1}^{N_S} deaths}{\sum_{n=1}^{N_S} person - years} * 100{,}000\right) - background\ mortality\ rate$$

For population strata only (i.e., ages were combined):

$$Crude\ mortality\ rate = \left(\frac{\sum deaths}{\sum person - years} * 100{,}000\right) - background\ mortality\ rate$$

$$Age - standardised\ mortality\ rate = \left(\frac{\sum_{n=1}^{N_{0-24y}} deaths}{\sum_{n=1}^{N_{0-24y}} person - years} * 47{,}000\right) + \left(\frac{\sum_{n=1}^{N_{25-44y}} deaths}{\sum_{n=1}^{N_{25-44y}} person - years} * 32{,}000\right) + \left(\frac{\sum_{n=1}^{N_{45-64y}} deaths}{\sum_{n=1}^{N_{45-64y}} person - years} * 21{,}000\right) - background\ mortality\ rate$$

3. Steps 1–2 were repeated for $b = 1000$ bootstrap samples, generating 1000 estimates within each strata
4. The quantile function was then used to return the 95% confidence intervals and point estimates for each rate as required $(0.025, 0.5, 0.975)$.

References

1. Davison A, Hinkley D. (1997). Bootstrap Methods and their Application (Cambridge Series in Statistical and Probabilistic Mathematics). Cambridge: Cambridge University Press. https://doi.org/10.1017/CBO9780511802843
2. Stacey I, Seth R, Nedkoff L, Hung J, Wade V, Haynes E, et al. Rheumatic heart disease mortality in Indigenous and non-Indigenous Australians between 2010 and 2017. Heart. 2023;109:1025–33. PubMed https://doi.org/10.1136/heartjnl-2022-322146
3. Katzenellenbogen JM, Bond-Smith D, Seth RJ, Dempsey K, Cannon J, Stacey I, et al. Contemporary incidence and prevalence of rheumatic fever and rheumatic heart disease in Australia using linked data: the case for policy change. J Am Heart Assoc. 2020;9:e016851. PubMed https://doi.org/10.1161/JAHA.120.016851

Appendix Table 1. ICD-10 Australian Modification (AM) codes for comorbidity identification

Comorbidity	ICD-10AM codes
Autoimmune diseases	M30-M35, K50-K52, L40, E06.3, E05.0, M45.9, K90.0, K75.4, K74.3, M30.0, L10.0, L95.9, M06.9, M34.0, M35.0, M35.3, D86.9, D59.0, D59.1, D69.2, D61.0, G70.0, M31.5, M31.30, M33.90, I73.00, D89.89
COPD	J40-J47
CKD	I12, I13, N00-N08, N11, N12, N14-N16, N18, N19, N25-N28, Q60-Q63, Z49, E10.2, E11.2, E12.2, E13.2, E14.2, I15.0, I15.1, N39.1, N39.2, T82.4, Z94.0, Z99.2
Hypertension	I10-I15
Diabetes	E10-E14
Ischaemic Heart Disease	I20 – I25
Stroke	I60-I64, I69
Heart failure	I50
Endocarditis	I33
Atrial fibrillation	I48
Chronic alcohol use	F10, K70, E24.4, G31.2, G62.1, G72.1, I42.6, K29.2, K85.2, K86.0, O35.4, R78.0, T51.0, T51.1, T51.9, Z50.2, Z71.4, Z72.1, Z86.41
Mental health (anxiety, depression and other mood disorders)	F40-F41, F32.0 - F32.2, F32.8, F32.9, F33.0, F33.2, F33.4 - F33.9, F34, F38, F39
Smoking	F17.1, F17.2, Z72.0, Z86.43

Appendix Table 2. Denominator information used in rate calculations

Population	Indigenous person-years	Non-Indigenous person-years
RHD study cohort		
0–24y	6,368.25	883.97
25–44y	9,044.36	3,961.00
45–64y	6,041.28	13,457.32
Residential population		
0–24y	1,871,571	25,362,518
25–44y	875,704	22,926,433
45–64y	566,478	20,261,551

Appendix Table 3. Baseline demographics for (A) all individuals comprising the RHD study cohort and (B) the subset of individuals who died within the RHD study cohort (*2*).

Demographic	(A) RHD study cohort, N (%)	(B) RHD study cohort, deaths only All N (%)	Indigenous N (%)	non-Indigenous N (%)
Total:	9342 (100)	726 (100)	401 (100)	325 (100)
Age at study entry:				
0–24 y	2301 (24.6)	26 (3.6)	21 (5.2)	5 (1.5)
25–44 y	3214 (34.4)	175 (24.1)	129 (32.2)	46 (14.2)
45–64 y	3827 (41.0)	525 (72.3)	251 (62.6)	274 (84.3)
Sex:				
Male	3218 (34.4)	300 (41.3)	170 (42.4)	130 (40.0)
Female	6124 (65.6)	426 (58.7)	231 (57.6)	195 (60.0)
Population:				
Indigenous	5193 (55.6)	401 (100)	401 (100)	-
Immigrant from low/middle income country	1786 (19.1)	117 (16.1)	-	117 (36.0)
Other Australian	2363 (25.3)	208 (64.0)	-	208 (64.0)
Location:				
Metropolitan resident	2987 (32.0)	288 (39.7)	56 (14.0)	232 (71.4)
Jurisdiction:				
Northern Territory	2625 (28.1)	211 (29.1)	191 (47.6)	20 (6.2)
South Australia	323 (3.5)	42 (5.8)	13 (3.2)	29 (8.9)
Queensland	2941 (31.5)	189 (26.0)	97 (24.2)	92 (28.3)
Western Australia	1067 (11.4)	88 (12.1)	54 (13.5)	34 (10.5)
New South Wales	2386 (25.5)	196 (27.0)	46 (11.5)	150 (46.2)
Hospitalizations before study entry:				
Ischaemic heart disease	1720 (18.4)	340 (46.8)	203 (50.6)	137 (42.2)
Atrial fibrillation	2849 (30.5)	366 (50.4)	199 (49.6)	167 (51.4)
Endocarditis	548 (5.9)	111 (15.3)	52 (13.0)	59 (18.2)

Demographic	(A) RHD study cohort, N (%)	(B) RHD study cohort, deaths only<br>All N (%)	Indigenous N (%)	non-Indigenous N (%)
Heart failure	2426 (26.0)	511 (70.4)	286 (71.3)	225 (69.2)
Stroke	621 (6.6)	130 (17.9)	65 (16.2)	65 (20.0)
Diabetes	1815 (19.4)	307 (42.3)	210 (52.4)	97 (29.8)
Hypertension	2214 (23.7)	422 (58.1)	266 (66.3)	156 (48.0)
Chronic kidney disease	1596 (17.1)	382 (52.6)	246 (61.3)	136 (41.8)
Chronic obstructive pulmonary disease	987 (10.6)	204 (28.1)	132 (32.9)	72 (22.2)
Autoimmune disease	491 (5.3)	90 (9.6)	56 (14.0)	34 (10.5)
Chronic alcohol-related hospitalization	1394 (14.9)	209 (28.8)	174 (43.4)	35 (10.8)
Smoking on hospitalization record	4771 (51.1)	209 (28.8)	314 (78.3)	195 (60.0)
Mental health-related hospitalization	554 (5.9)	91 (12.5)	36 (9.0)	55 (16.9)

Appendix Table 4. Expected rheumatic heart disease (RHD)-related deaths compared to Australian Bureau of Statistics (ABS) RHD-coded deaths for Australians aged <65 y, 2013–2017 (frequencies, differences and proportions)

	Indigenous				Non-Indigenous			
Patient age	**Expected***	**ABS RHD-coded†**	**Difference (Coded-Expected)**	**Proportion (Coded/ Expected)**	**Expected***	**ABS RHD-coded†**	**Difference (Coded-Expected)**	**Proportion (Coded/ Expected)**
All ages	319	145	−174	46%	272	300	+28	110%
0–24 y	9	8	−1	89%	<5	7		
25–44 y	88	48	−40	55%	29	41	+12	141%
45–64 y	222	89	−133	40%	240	252	+12	105%

*Expected RHD-related deaths, calculated based on excess mortality rates in Table 1 applied to person-years within matching age and population strata. ABS RHD-coded deaths (RHD as an underlying or associated cause of death).

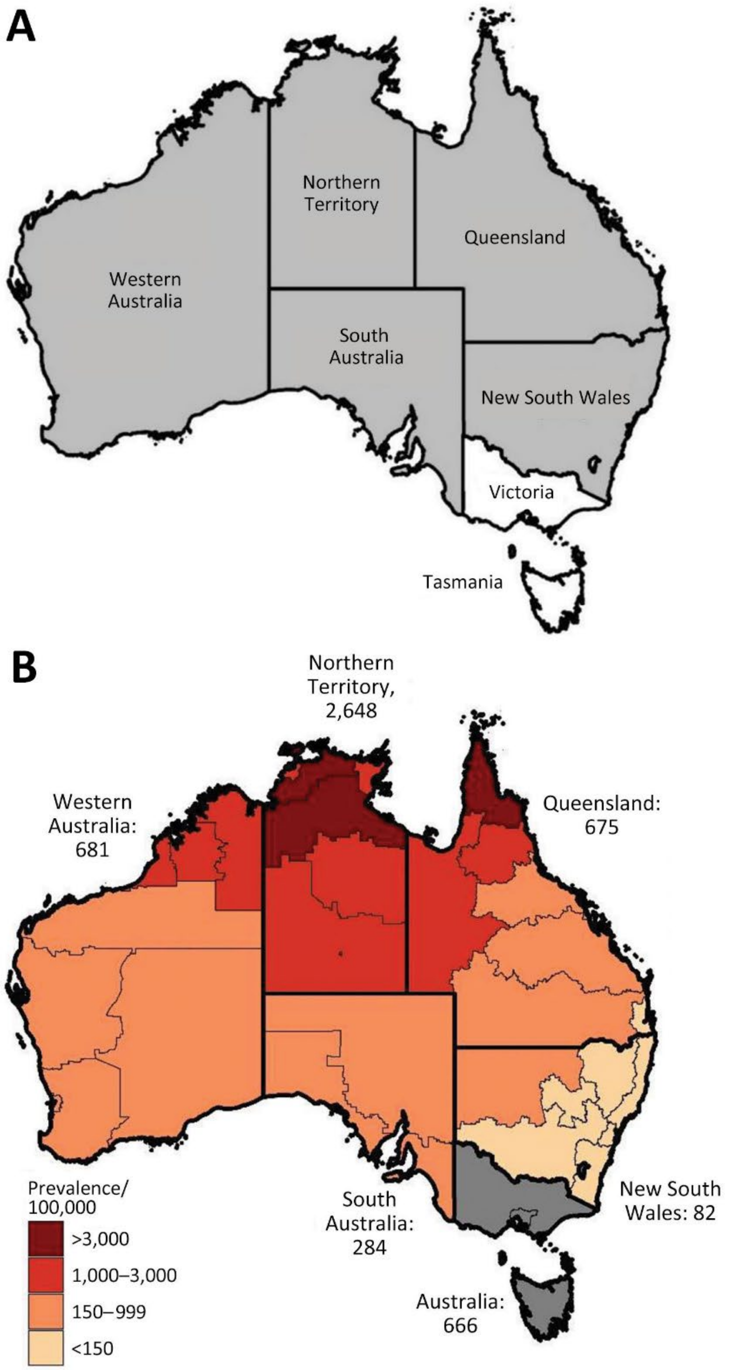

Appendix Figure. Upper map: The five Australian jurisdictions that were included in the End-RHD in Australia: Study of Epidemiology (ERASE) project are highlighted in grey. These jurisdictions are home to 71% of the total general population and 86% of the Aboriginal and Torres Strait Islander (Indigenous) population. Lower map: Age-standardised RHD prevalence per 100,000 person-years in the Indigenous Australian population, generated by the ERASE project (*3*).
